# Potentilloside A, a New Flavonol-*bis*-Glucuronide from the Leaves of *Potentilla chinensis*, Inhibits TNF-α-Induced ROS Generation and MMP-1 Secretion

**DOI:** 10.3390/plants11233318

**Published:** 2022-12-01

**Authors:** So Young Lee, Yea Jung Choi, So-Ri Son, Young-Seo Yoon, Sun-Hee Lee, Kyung-Tae Lee, Sullim Lee, Dae Sik Jang

**Affiliations:** 1 Department of Biomedical and Pharmaceutical Sciences, Graduate School, Kyung Hee University, Seoul 02447, Republic of Korea; 2 College of Korean Medicine, Gachon University, Seongnam 13120, Republic of Korea; 3 Department of Pharmaceutical Biochemistry, College of Pharmacy, Kyung Hee University, Seoul 02447, Republic of Korea; 4 Department of New Material Development, COSMAXBIO, Seongnam 13486, Republic of Korea; 5 Department of Life Science, College of Bio-Nano Technology, Gachon University, Seongnam 13120, Republic of Korea

**Keywords:** *Potentila chinensis*, Rosaceae, flavonol-*bis*-glucuronide, potentilloside A, reactive oxygen species, matrix metalloproteinase-1

## Abstract

The major contributor to skin aging is UV radiation, which activates pro-inflammatory cytokines including TNF-α. TNF-α is involved in the acceleration of skin aging via ROS generation and MMP-1 secretion. In our preliminary study, a 30% EtOH extract from the leaves of *Potentilla chinensis* (LPCE) significantly inhibited TNF-α-induced ROS generation in human dermal fibroblasts (HDFs). Therefore, the objective of this study is to identify the active components in LPCE. A new flavonol-*bis*-glucuronide (potentilloside A, **1**) and 14 known compounds (**2**–**15**) were isolated from an LPCE by repeated chromatography. The chemical structure of the new compound **1** was determined by analyzing its spectroscopic data (NMR and HRMS) and by acidic hydrolysis. Nine flavonols (**2**–**9** and **11**) and two flavone glycosides (**12** and **13**) from *P. chinensis* were reported for the first time in this study. Next, we evaluated the effects of the isolates (**1**–**15**) on TNF-α-induced ROS generation in HDFs. As a result, all compounds significantly inhibited ROS generation. Furthermore, LPCE and potentilloside A (**1**) remarkably suppressed MMP-1 secretion in HDFs stimulated by TNF-α. The data suggested that LPCE and potentilloside A (**1**) are worthy of further experiments for their potential as anti-skin aging agents.

## 1. Introduction

*Potentilla chinensis* Ser., belonging to the family Rosaceae, is widely distributed in East Asia—particularly in China, Japan, and Korea [1]. The aerial parts of *P. chinensis* (Potentillae Herba) have been used in Korea as an herbal medicine against various diseases, including dysentery, hemoptysis, colitis, etc. [2]. Pharmacological studies on *P. chinensis* have shown that it has antioxidant, inflammatory, hypoglycemic, anticancer, and immunomodulatory properties [3,4]. Phytochemical studies on *P. chinensis* have revealed the presence of triterpenoids, flavonoids, organic acids, and phenolic compounds [5,6]. Triterpenoids from *P. chinensis* have been shown to have anticancer, anti-inflammatory, neuroprotection, and hepatic injury attenuating effects [7,8,9,10]. Tiliroside—a flavonoid from *P. chinensis*—possesses antioxidant, anti-obesity, and antidiabetic properties [11,12,13]. However, the chemical constituents of *P. chinensis* and their pharmacological effects are still poorly understood.

Skin aging is triggered by internal and external factors. Internal factors that change gene expression or the neuroendocrine system result in chronological aging [14]. External factors affect the outermost skin area, which is directly affected by environmental factors such as UV radiation and air pollution [15]. UV radiation critically damages the dermal layer, which provokes pro-inflammatory cytokines that lead to photoaging.

These aging factors cause the degradation of extracellular matrix (ECM) components, including collagens, elastins, and proteoglycans [16]. These components are secreted by fibroblasts to maintain skin strength, flexibility, and hydration [17]. In contrast, the degradation of the ECM causes skin aging, which manifests as wrinkles and hyperpigmentation [18,19]. 

Tumor necrosis factor-α (TNF-α), one of the pro-inflammatory cytokines, generates reactive oxygen species (ROS), which accelerate skin aging through the release of interleukin-1 (IL-1) and IL-6 [20,21]. In addition, TNF-α activates the AP-1 transcription pathway by stimulating mitogen-activated protein kinases (MAPKs) [22]. Consequentially, these result in overexpression of the following matrix metalloproteinases (MMPs); MMP-1, MMP-3, and MMP-9 [23]. MMP-1, a type of collagenase, accelerates skin aging by destroying collagen fiber to produce winkles [24]. Moreover, TNF-α also inhibits the transforming growth factor-β (TGF-β) pathway, which suppresses collagen synthesis and accelerates MMP secretion via ROS generation [25]. Thus, controlling TNF-α activity could be an efficient strategy for the development of anti-aging skin therapies. 

In our preliminary study, a 30% EtOH extract from the leaves of *P. chinensis* (LPCE) significantly inhibited ROS generation in TNF-α-induced human dermal fibroblasts (HDFs). Therefore, the purpose of the study is to identify the active isolates in the LPCE. Herein, the chemical constituents from an LPCE were isolated by repeated chromatography. The chemical structures of all the isolates were determined by interpreting their spectroscopic data, including 1D- and 2D-nuclear magnetic resonances (NMR) and high-resolution mass spectroscopy (HR-MS). Next, all isolates were assessed for their effects on ROS generation and MMP-1 secretion in TNF-α-stimulated HDFs.

## 2. Results

### 2.1. Structure Elucidation of ***1*** and Identification of ***2***–***15***

**Figure 1:**
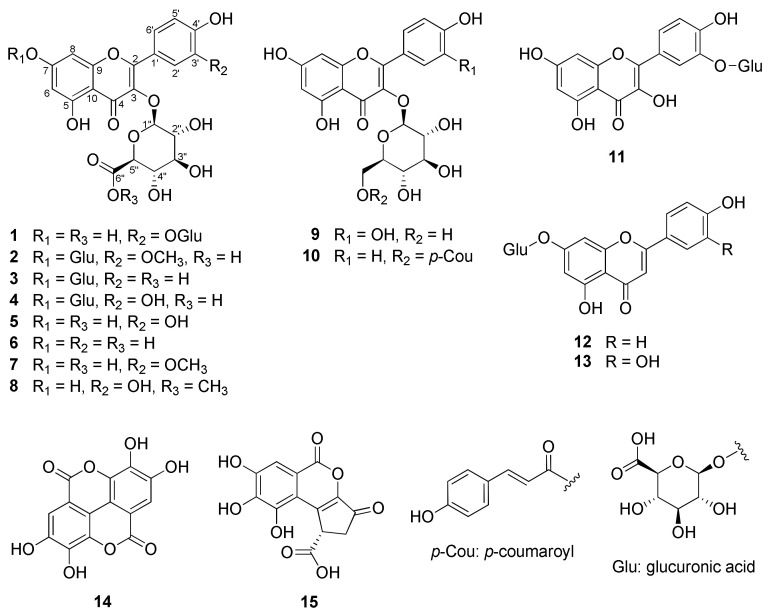
Structures of compounds **1**–**15** isolated from the leaves of *P. chinensis*.

In the present study, a new flavonol-*bis*-glucuronide (**1**)—along with the previously described ten flavonols (**2**–**11**), two flavones (**12** and **13**), and two polyphenols (**14** and **15**)—were isolated from the leaves of *P. chinensis* (Figure 1).

**Figure 2:**
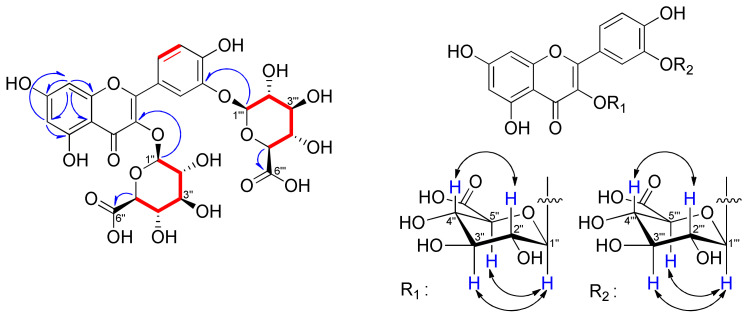
Key ^1^H−^1^H COSY (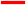), ^1^H−^13^C HMBC (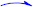) and ^1^H−^1^H NOESY (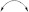) correlations of compound **1**.

Compound **1** was obtained as a yellow powder. The HR-ESI-Orbitrap-MS displayed a [M + H]^+^ peak at *m*/*z* 655.1141 (cal. for C_27_H_27_O_19_, 655.1147), corresponding to the molecular formula C_27_H_26_O_19_ (Figure S1). The infrared (IR) spectrum showed the presence of a hydroxy group (3395.07 cm^−1^), a conjugated carbonyl (1792.83 cm^−1^), and an aromatic ring (1653.66, 1603.52, and 1503.24 cm^−1^; Figure S2). The ^1^H NMR spectrum of **1** exhibited the characteristic signals of a flavonol glycoside (Table 1 and Figure S3). The aglycone was identified as quercetin from three aromatic proton signals for an ABX spin system belonging to ring B (*δ*_H_ 6.92 (1H, d, *J* = 8.5 Hz, H-5′), 7.75 (1H, d, *J* = 2.5 Hz, H-2′) and 7.89 (1H, dd, *J* = 8.5, 2.5 Hz, H-6′)) and two aromatic signals for an AX spin system belonging to ring A (*δ*_H_ 6.23 (1H, d, *J* = 2.0 Hz, H-6) and 6.48 (1H, d, *J* = 2.0 Hz, H-8)). Additionally, two anomeric proton signals (*δ*_H_ 5.46 (1H, d, *J* = 7.5 Hz, H-Glu-1″) and 5.08 (1H, d, *J* = 7.5 Hz, H-Glu-1‴)) were observed (Table 1). The ^13^C and HSQC NMR spectra of **1** revealed 27 characteristic carbon signals and indicated the presence of five aromatic methine and ten quaternary carbons, including one carbonyl (*δ*_C_ 177.3) and seven oxygenated carbons (Table 1, Figures S4 and S5). Additionally, four carbon signals at *δ*_C_ 101.1, 101.5, 170.1, and 170.3 and eight carbon signals at *δ*_C_ 70–80 implicated the existence of two glucuronic acids in **1**. The ^1^H and ^13^C NMR spectroscopic data of **1** were very similar to quercetin-3-*O*-*β*-d-glucuronide (**5**), except for the presence of one additional glucuronic acid (Table 1). Using the COSY spectroscopic data, correlations in the A and B rings as well as in the sugars were observed, confirming their connections (Figure 2 and Figure S6). The locations of the two glucuronic acids were determined to be C-3 and C-3′ by the HMBC data from H-1″ to C-3 and H-1‴ to C-3′ (Figure 2 and Figure S7). The relative-configurations of the sugars were determined by the *J* value of 7.5 Hz for the anomeric protons and by correlations observed in the ROESY spectrum between H-1″ and H-3″ and H-5″/H-1‴ and H-3‴ and H-5‴ (Figure 2 and Figure S8). In addition, the absolute configurations of the glucuronic acids were confirmed as d-forms through HPLC analysis after sugar hydrolysis. Therefore, the chemical structure of the new flavonol glycoside **1** was elucidated as quercetin-*bis*-3,3′-*O*-*β*-d-glucuronide, named potentilloside A.

By comparing the spectroscopic data with those reported in the literature, the known compounds were identified as isorhamnetin-*bis*-3,7-*O*-*β*-d-glucuronide (**2**), kaempferol-*bis*-3,7-*O*-*β*-d-glucuronide (**3**), quercetin-*bis*-3,7-*O*-*β*-d-glucuronide (**4**) [26,27] quercetin-3-*O*-*β*-d-glucuronide (**5**) [28], kaempferol-3-*O*-*β*-d-glucuronide (**6**) [29], isorhamnetin-3-*O*-*β*-d-glucuronide (**7**) [30], quercetin-3-*O*-*β*-d-glucuronide-6″-methyl ester (**8**) [31], isoquercitrin (**9**) [32], tiliroside (**10**) [33], quercetin-3′-*O*-*β*-d-glucuronide (**11**) [34], apigenin-7-*O*-*β*-d-glucuronide (**12**) [35], luteolin-7-*O*-*β*-d-glucuronide (**13**) [35], ellagic acid (**14**) [36], and brevifolin carboxylic acid (**15**) [37].

### 2.2. Effects of LPCE and Isolates ***1***–***15*** on HDF Cell Viability

**Figure 3:**
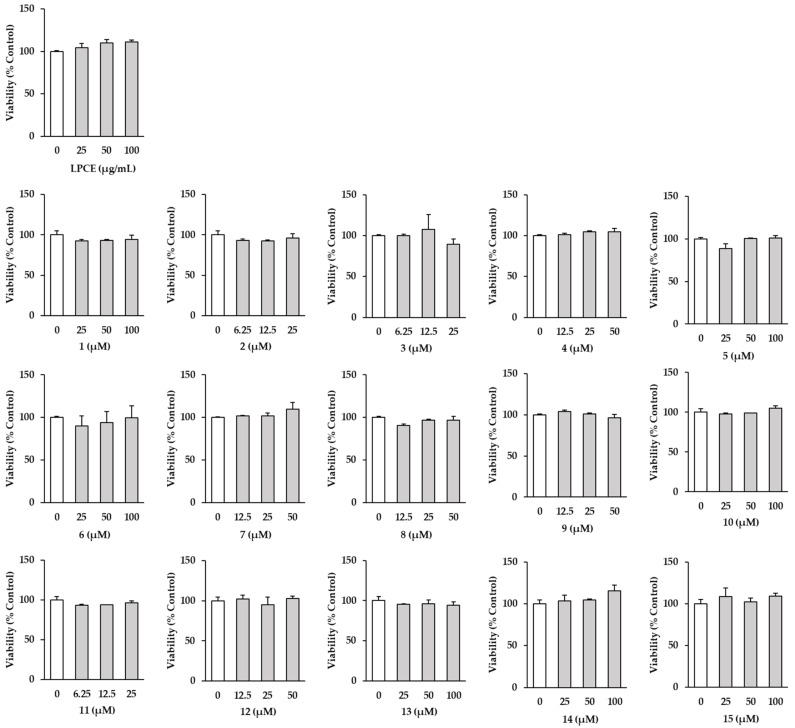
Effect of the extract (LPCE) and isolates (**1**–**15**) from *P. chinensis* on cell viability in HDFs. HDF cells were seeded in a 96-well cell culture plate at a concentration of 0.5 × 10^4^/well and incubated in starvation conditions with fresh media without FBS for 24 h. Then, the cells were treated with specific concentrations of LPCE and isolates for 24 h. The resulting values were determined using an EZ-Cytox solution Assay kit. The data are presented as mean ± SEM (*n* = 2).

Before evaluating the effects of LPCE and its isolated compounds on TNF-α-induced HDFs, an experiment was performed to find the non-effect range on cell viability. A LPCE showed no change in cell viability at concentrations of 25–100 µg/mL. Additionally, none of the isolates showed changes in cell viability at the indicated concentrations in Figure 3. Therefore, subsequent experiments were performed at each concentration.

### 2.3. Effects of LPCE and Isolates ***1***–***15*** on TNF-α-Induced ROS Generation

**Figure 4:**
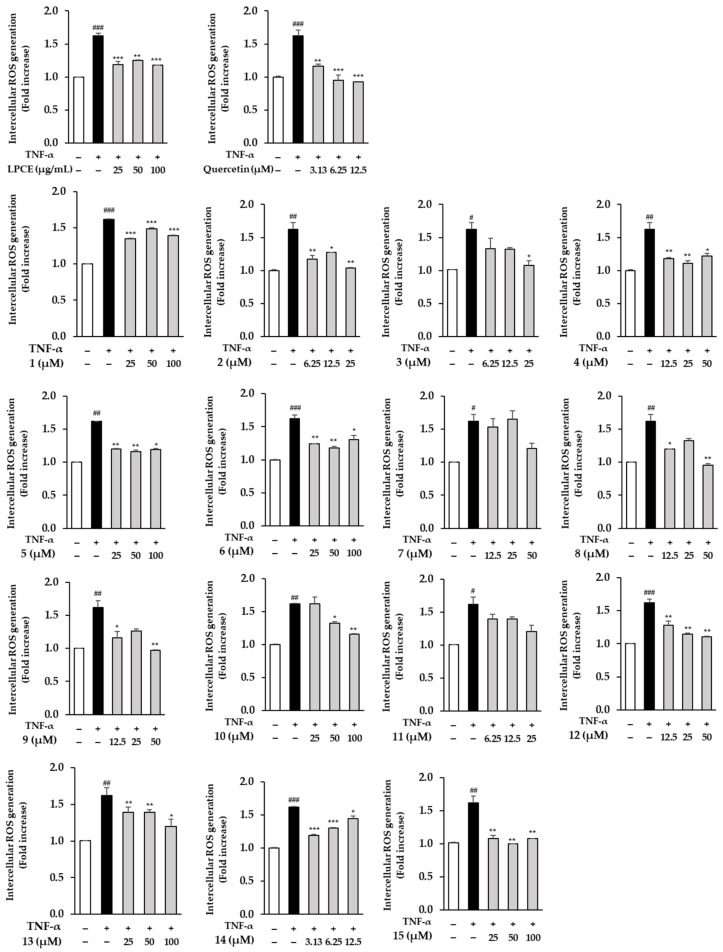
Inhibitory effect of the extract (LPCE) and isolates (**1**–**15**) from *P. chinensis* on intracellular ROS generation in TNF-α-induced HDFs. The HDF cells were plated at a concentration of 1 × 10^4^ cells/well in a black 96-well plate and incubated for 24 h. The previous media were exchanged with fresh media without FBS for 24 h for cell cycle arrest. After that, LPCE and isolates were treated with the indicated concentration for 1 h, and continuously cotreated with 20 ng/mL of TNF-α and 10 µM of the probe dichlorofluorescin diacetate (DCFDA) for 15 min. The data are presented as mean ± SEM (*n* = 2). ### *p* < 0.001, ## *p* < 0.01 and # *p* < 0.05 compared with the untreated group; *** *p* < 0.001, ** *p* < 0.01 and * *p* < 0.05 compared with the TNF-α-treated group. Quercetin was used as a positive control.

Subsequently, the inhibitory effects of the LPCE and all the *P. chinensis* isolates on ROS generation were tested in TNF-α-stimulated HDFs. At the concentrations shown in Figure 4, the LPCE and all the isolates inhibited ROS production. Specifically, potentilloside A (**1**) suppressed the level of ROS at 25 µM (1.35 ± 0.00-fold, *p* < 0.001), 50 µM (1.49 ± 0.01-fold, *p* < 0.001), and 100 µM (1.39 ± 0.01-fold, *p* < 0.001) in comparison to the TNF-α-treated group (1.62 ± 0.00-fold, *p* < 0.001). Additionally, two flavonol-*bis*-glucuronides—isorhamnetin-*bis*-3,7-*O*-*β*-d-glucuronide (**2**) and kaempferol-*bis*-3,7-*O*-*β*-d-glucuronide (**3**)—exhibited strong inhibitory effects on ROS generation at 25 µM in comparison to the TNF-α-treated group. Additionally, quercetin inhibited ROS generation to 3.13 µM (1.17 ± 0.03, *p* < 0.01), 6.25 µM (1.17 ± 0.03, *p* < 0.01), and 12.5 µM (1.17 ± 0.03, *p* < 0.01) in comparison with the TNF-α- treated group (1.62 ± 0.09, *p* < 0.001).

### 2.4. Effects of LPCE and Flavonol-bis-Glucuronides ***1***–***4*** on TNF-α-Induced MMP-1 Secretion

**Figure 5:**
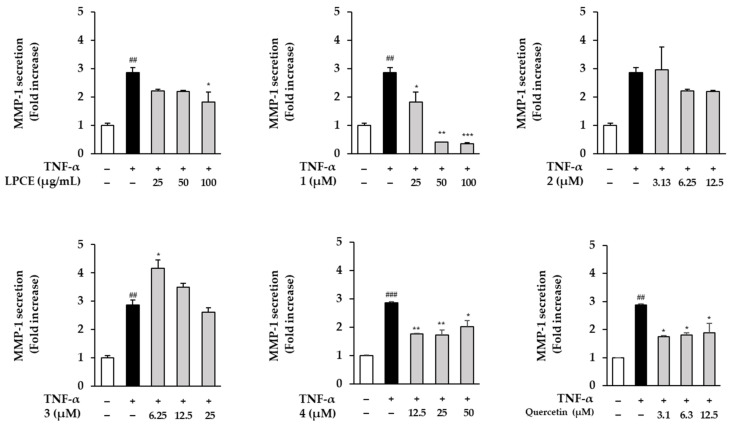
Effect of LPCE and compounds **1**–**4** from *P. chinensis* on MMP-1 protein expression in TNF-α-stimulated HDFs. The HDF cells were plated at a concentration of 1 × 10^4^ cells/well in a black 96-well plate and incubated for 24 h. The previous media were exchanged with fresh media without FBS for 24 h for cell cycle arrest. After that, the extract and isolates were treated with the indicated concentration for 1 h, and continuously treated with 20 ng/mL of TNF-α for 24 h. The MMP-1 secretion levels were measured using an ELISA kit. The data are presented as mean ± SEM (*n* = 2). ### *p* < 0.001 and ## *p* < 0.01 compared with the untreated group; *** *p* < 0.001, ** *p* < 0.01, and * *p* < 0.05 compared with the TNF-α-treated group. Quercetin was used as a positive control.

The various types of isolates from *P. chinensis* exhibited inhibitory effects on the generation of ROS in TNF-α-induced HDFs in this study. Among them, flavonol-*bis*-glucuronides are rare in nature and their pharmacological activities are little-known. Thus, compounds **1**–**4** were selected to evaluate their inhibitory effects on MMP-1 secretion in HDFs stimulated by TNF-α. As shown in Figure 5, LPCE and flavonol-*bis*-glucuronides **1**–**4** inhibited MMP-1 secretion of TNF-α-induced HDFs. The TNF-α-treated group increased MMP-1 secretion to 2.87 ± 0.17-fold (*p* < 0.01) in comparison to the vehicle group. LCPE decreased MMP-1 secretion with all dosages; notably, at the concentration of 100 μg/mL, MMP-1 secretion decreased to 1.83 ± 0.35-fold (*p* < 0.05) in comparison with the TNF-α-treated group. Potentilloside A (**1**) inhibited MMP-1 secretion at 50 μM (0.42 ± 0.00-fold, *p* < 0.001) and 100 μM (0.35 ± 0.03-fold, *p* < 0.001) in comparison to the TNF-α-treated group. In addition, quercetin-*bis*-3,7-*O*-*β*-d-glucuronide (**4**) also significantly diminished MMP-1 secretion at 12.5–50 μM (12.5 μM: 1.76 ± 0.02-fold, *p* < 0.01; 25 μM: 1.73 ± 0.17-fold, *p* < 0.01; 100 μM: 2.02 ± 0.21-fold, *p* < 0.05) in contrast to TNF-α-treated group (2.87 ± 0.03, *p* < 0.001). In the case of the positive control, the TNF-α-treated group significantly increased MMP-1 secretion (2.87 ± 0.05-fold, *p* < 0.01) in comparison with the vehicle control group. Quercetin decreased MMP-1 secretion at 3.1 μM (1.75 ± 0.05, *p* < 0.05), 6.3 μM (1.82 ± 0.07, *p* < 0.05), and 12.5 μM (1.90 ± 0.32, *p* < 0.05) in comparison with the TNF-α-treated group.

## 3. Materials and Methods

### 3.1. General Experimental Procedures

Thin layer chromatography (TLC) analyses were performed on Silica gel 60 F254 (Merck, MA, USA) and RP-18 F254S (Merck, MA, USA) plates. After TLC development in a confirmed solvent system, TLC plates were charred by 20% (*v*/*v*) H_2_SO_4_ reagent (Duksan, Seoul, Republic of Korea) and then heated at 123 °C for 10 min. UV spectra were obtained with a Perkin Elmer Lambda 35 UV/VIS spectrometer (PerkinElmer, Shelton, CT, USA). Optical rotations were obtained on a Jasco P-2000 polarimeter (JASCO, Tokyo, Japan), using a 10 mm microcell. JEOL (JEOL, Tokyo, Japan) 500 MHz was used for obtaining NMR spectra. HR-ESI-Orbitrap-MS spectra were obtained using an LTQ-Orbitrap mass spectrometer (Thermo scientific, Waltham, MA, USA). Agilent Cary 630 FT-IR (Agilent Technologies, Santa Clara, CA, USA) was applied to obtain the IR spectrum. Sephadex LH-20 (Merck, MA, USA), Silica gel (230–400 mesh and 70–230 mesh, Merck, MA, USA), and Diaion HP-20 (Mitsubishi, Tokyo, Japan) were used for open column chromatography. Pre-packed cartridges, Redi Sep-Silica (12 g, 24 g, 40 g, 80 g, and 120 g, Teledyne Isco, Lincoln, NE, USA) and Redi Sep-C18 (13 g, 26 g, 43 g, and 130 g, Teledyne Isco, Lincoln, NE, USA) were used for flash chromatography. Prep HPLC was performed using a Waters purification system (Waters corporation, MA, USA) equipped with a 1525 pump, PDA 1996 detector, and Gemini NX-C18 110A column (250.0 × 21.2 mm i.d., 5.0 μm, Phenomenex, CA, USA).

### 3.2. Plant Material

The dried leaves of *Potentilla chinensis* Ser. (Rosaceae) were obtained from COSMAX BIO (Seongnam-si, Republic of Korea) in April 2021 and were identified by Professor Dae Sik Jang. A voucher specimen (POCH-2021) has been deposited in the herbarium of the college of Pharmacy, Kyung Hee University, Seoul, Republic of Korea.

### 3.3. Extraction and Isolation

The ground plant materials (3 kg) were extracted with 75 kg of 30% EtOH using a pilot extraction system (60 °C, 5 h). The extracts were filtered and then concentrated under reduced pressure at 45 °C to obtain a 30% EtOH extract (LPCE, 351.45 g). 

A portion of the EtOH extract (35.15 g) was fractionated using Diaion HP-20 (*ϕ* 4.8 × 35.0 cm) column chromatography (CC) with a gradient system (MeOH/H_2_O = 0:1 to 1:0, *v*/*v*) to afford ten fractions (F1–F10). F2 (3.42 g) was fractionated by Sephadex LH-20 CC (*ϕ* 3.6 × 68.0 cm, 50% MeOH) to obtain nine subfractions (F2-1–F2-9). F2-2 (1.0 g) was subjected to reverse phased CC (*ϕ* 3.5 × 35.0 cm MeOH with 0.1% TFA/H_2_O with 0.1% TFA = 10:90 to 0:1, *v*/*v*) to give compounds **2** (6.4 mg) and **4** (14.8 mg). F2-3 (540 mg) was separated by flash CC (Redi Sep-RP cartridge 130 g, MeOH with 0.1% formic acid (FA)/H_2_O with 0.1% FA = 20:80 to 30:70, *v*/*v*) to give compound **1** (68.3 mg). F6 (1.74 g) was subjected to Sephadex LH-20 CC (*ϕ* 3.4 × 72.0 cm, MeOH/H_2_O = 1:0 to 80:20, *v*/*v*) to afford six subfractions (F6-1–F6-6). F6-5 (482.0 mg) was separated further by flash CC (Redi Sep-silica cartridge 80 g, CH_2_Cl_2_/90% MeOH = 90:10 to 60:40, *v*/*v*) to isolate compound **5** (29.3 mg). F9 (767.2 mg) was separated by Sephadex LH-20 CC (*ϕ* 2.5 × 62.5 cm, MeOH) to obtain seven subfractions (F9-1–F9-7). Compound **14** (30.0 mg) was purified by recrystallization in MeOH from F9-7 (67.8 mg). Compound **10** (11.2 mg) was isolated from F9-5 (42.5 mg) using flash CC (Redi Sep-RP cartridge 43 g, MeOH/H_2_O = 50:50 to 70:30, *v*/*v*).

The remaining 30% EtOH extract (316.3 g) was chromatographed over a Diaion HP-20 (*ϕ* 8.5 × 54.0 cm), eluting with MeOH-H_2_O (from 0:1 to 1:0, *v*/*v*) to afford seven fractions (R1–R7). R4 (5.0 g) was fractionated further by Sephadex LH-20 CC (*ϕ* 4.5 × 57 cm, MeOH/H_2_O = 40:60 to 1:0, *v*/*v*) to obtain six subfractions (R4-1–R4-6). R4-2 (3.36 g) was separated by silica gel CC (230–400 mesh, *ϕ* 4.5 × 28.0 cm, CH_2_Cl_2_:90% MeOH = 70:30 to 0:1 *v*/*v*) to obtain compound **3** (7.4 mg). Compound **15** (78.2 mg) was purified by flash CC (Redi Sep-RP cartridge 43 g, MeOH with 0.1% FA/H_2_O with 0.1% FA = 10:90 to 25:75, *v*/*v*) from R4-5 (134.3 mg). R6 (28.3 g) was fractionated into eight subfractions (R6-1–R6-8) by Sephadex LH-20 CC (*ϕ* 5.0 × 55.0 cm, MeOH/H_2_O = 80:20 to 1:0, *v*/*v*). R6-5 (2.0 g) was separated into seven subfractions (R6-5-1–R6-5-7) by silica gel CC (230–400 mesh; *ϕ* 4.0 × 24.0 cm; CH_2_Cl_2_/90% MeOH = 80:20 to 70:30, *v*/*v*). Compound **12** (1.0 mg) was obtained from R6-5-6 (159.3 mg) by reversed-phase MPLC with a Redi Sep-C18 cartridge (50 g, MeOH-H_2_O, from 10:90 to 40:60, *v*/*v*). Compound **13** (4.3 mg) was purified from R6-5-7 (230.0 mg) by reversed-phase MPLC with a Redi Sep-C18 cartridge (50 g, MeOH-H_2_O, from 10:90 to 40:60, *v*/*v*), followed by preparative HPLC on a Gemini NX-C18 110A column (MeOH with 0.1% FA-H_2_O with 0.1% FA, 40:60, *v*/*v*). R6-7 (2.0 g) was chromatographed over a Silica gel (230–400 mesh; *ϕ* 4.0 × 22.0 cm), eluting with CH_2_Cl_2_/90% MeOH (from 70:30 to 0:1, *v*/*v*) to afford nine subfractions (R6-7-1–R6-7-9). Compounds **8** (1.0 mg) and **9** (8.4 mg) were obtained from R6-7-3 (49.0 mg) and R6-7-4 (100.0 mg), respectively, by prep HPLC column (Gemini NX-C18 110A, MeOH with 0.1% FA/H_2_O with 0.1% FA = 45:55, *v*/*v*). Subfraction R6-7-7(320.0 mg) was separated further by flash CC with a Redi Sep-silica cartridge (40 g, CH_2_Cl_2_/90% MeOH = 80:20 to 65:35, *v*/*v*) to give six subfractions (R6-7-7-1–R6-7-7-6). Subfraction R6-7-7-4 (2.4 g), using a flash chromatographic system with a Redi Sep-RP cartridge (130 g, acetonitrile/H_2_O = 60:40 to 75:25, *v*/*v*), was used to obtain five subfractions (R6-7-7-4-1~R6-7-7-4-5). Compounds **6** (2.5 mg), **7** (4.2 mg), and **11** (10.4 mg) were obtained from R6-7-7-4 (47.4 mg), followed by preparative HPLC on a Gemini NX-C18 110A column (MeOH with 0.1% FA/H_2_O with 0.1% FA, from 22:78 to 23:77, *v*/*v*). 

#### Potentilloside A (**1**)

Yellow powder; m.p.: 174.1 °C; [α]_D_^22^: −31.6 (c 0.1, H_2_O); UV (H_2_O) λ_max_ (log ε) 264 (4.02), 346 nm (3.97); IR (ATR) ν_max_ 3395.07, 1792.83, 1653.66, 1603.52, 1503.24 cm^−1^; HR-ESI-Orbitrap-MS (positive mode) *m*/*z* = 655.1141 [M + H]^+^ (calcd for C_27_H_27_O_19_, 655.1147); ^1^H-NMR (DMSO-*d*_6_, 500 MHz) and ^13^C-NMR (DMSO-*d*_6_, 125 MHz) data, see Table 1. 

### 3.4. Acidic Hydrolysis of **1** and Sugar Identification

The absolute configuration of the glucuronic acid moiety in compound **1** was determined as in reference [38]. Compound **1** (1.0 mg) was hydrolyzed in 1% HCl (1 mL) at 100 °C for 24 h. The hydrolysate was dissolved in pyridine (500 µL) and l-cysteine methyl ester hydrochloride (1.2 mg) was added and heated at 60 °C for 1 h. σ-Tolyl isothiocyanate (100 µL) was added and heated again at 60 °C for 1 h. The glucuronic acid in the reaction mixture of **1** was detected at 20.74 min by HPLC under a gradient system (Mobile phase MeCN−H_2_O (25:75, *v*/*v*) containing 50 mM H_3_PO_4,_ 30 min). The retention time of the authentic d-glucuronic acid was 20.77 min under the same HPLC conditions. Therefore, the absolute configuration of *β*-glucuronic acid in **1** was confirmed as the d-configuration.

### 3.5. Cell Culture Conditions

HDFs were purchased from PromoCell (Sickingenstr, Heidelberg, Germany). The cells were cultured in Dulbecco’s modified Eagle’s medium (Gibco, GrandIsland, NY, USA) supplemented with 10% fetal bovine serum (Atlas, Fort Collins, CO, USA) and penicillin–streptomycin solution (Welegen, Seoul, Republic of Korea). Cells were incubated in a humid cell incubator (Thermo Scientific, Waltham, MA, USA) at 37 °C in a 5% CO_2_ atmosphere. 

### 3.6. Sample Preparations

The extract and compounds **1**–**15** were dissolved in DMSO (Sigma-Aldrich, St. Louis, MO, USA) to 10 mM. The final concentration of DMSO was kept below 1% for each sample treatment. TNF-α (PeproTech, Rocky Hill, NJ, USA) was dissolved in 1% bovine serum albumin (ROCHE, Basel, Switzerland) solution to obtain 20 µg/mL and stored at −20 °C until use. 

### 3.7. Cell Viability

HDFs were plated in 96-well cell culture plates at a concentration of 5 × 10^3^ cells/well and incubated in a humid cell incubator for 24 h. After this, the extract and compounds were treated with the attached cells at the indicated concentration for 24 h. Next, 100 μL of 10% Ez-cytox solution was added to each well to evaluate the cytotoxicity of the extract and isolates on HDFs. The values were deterimined using a SPARK 10M (Tecan Group Ltd., Männedorf, Switzerland). 

### 3.8. ROS Generation Assay

HDFs were seeded in 96-well black plates at a concentration of 1 × 10^4^ cells/well and incubated in a humid cell incubator for 24 h; then, the media was exchanged with new media without FBS to arrest the cell cycle. Next, the plated cells were treated with the extract and compounds at the indicated concentrations for 1h. After that, 20 ng/mL of TNF-α (PeproTech) was continuously added to each well and incubated. After 15 min of incubation, the probe dichlorofluorescein diacetate (DCFDA; Sigma-Aldrich) was treated at a concentration of 10 μM and each well washed with 100 μL of phosphate-buffered saline (PBS; Welgene, Gyeongsangbuk, Republic of Korea). Fluorescence was measured using a SPARK 10M, and the wavelength was set to 485/535 nm. The results for the intracellular ROS levels are presented as a percentage of the vehicle control.

### 3.9. Enzyme-Linked Immunosorbent Assay (ELISA)

HDFs were seeded in 48-well cell culture plates at a concentration of 2 × 10^4^ cells/well for 24 h. Then, the previous media was replaced with fresh media without FBS to arrest the cell cycle. Next, the extract and compounds were treated at a specific concentration for 1 h. Then, 20 ng/mL of TNF-α was added to each well for 24 h. After this, the supernatants were collected to measure MMP-1 secretion using an ELISA kit. The results were determined using a SPARK 10M.

### 3.10. Statistical Analysis

Experimental data are presented mean ± standard error of the mean (SEM). The statistical analysis of the experimental results was conducted using a one-way ANOVA in GraphPad Prism software. The difference between each group was evaluated using Tukey’s multiple comparison at the range of *p* < 0.05.

## 4. Discussion

A new flavonol glycoside (potentilloside A, **1**) and 14 known compounds (**2**–**15**) were isolated from a 30% EtOH extract from the leaves of *P. chinensis* using repeated chromatography in the present study. Two flavonol-*bis*-glucuronides—isorhamnetin-*bis*-3,7-*O*-*β*-d-glucuronide (**2**) and kaempferol-*bis*-3,7-*O*-*β*-d-glucuronide (**3**)—were first isolated from *Geum rivale* L (Rosaceae) in 2021 [26]. The presence of quercetin-bis-3,7-*O*-*β*-d-glucuronide (**4**) was already identified from *Potentilla reptans* (Rosaceae), but its biological activities have not yet been reported [26,27]. Although the flavonol or flavone glycosides **5**–**13**, except for **10**, were all found in the same Rosaceae plants, they were first reported as constituents of *P. chinensis* in this study. Flavonoid glucuronides are rare in nature compared to other types of flavonoid glycosides. To the best of our knowledge, 211 kinds of flavonoid glucuronides have been reported to date. Among them, flavonoid *bis*-glucuronides represent only 22 kinds—including compounds **2**–**4** [39]. Thus, the biological activity of flavonoid glucuronides alone is not sufficient, although some of them have shown various pharmacological effects. For example, quercetin-3-*O*-*β*-d-glucuronide (**5**)—one of the most studied flavonoid glucuronides—exhibits antioxidant [40,41], anti-inflammatory [42], anticancer [43], amyloid β inhibitory [44], and anti-HIV activities [45]. Kaempferol-3-*O*-*β*-d-glucuronide (**6**) has also shown antioxidant [41], amyloid β inhibitory [44], and antibacterial effects [46]. In contrast, two flavone glucuronides—apigenin-7-*O*-*β*-d-glucuronide (**12**) and luteolin-7-*O*-*β*-d-glucuronide (**13**)—have displayed anti-inflammatory effects [47,48], vascular protective effects [48], and antigonadotrophic activities [49]. In addition, research on the various pharmacological activities of flavonoids is in progress. Since flavonoids are pleiotropic substances, it is difficult to evaluate their pharmacological and therapeutic potentials with just a few bioassays. Therefore, multifaceted approaches are needed to understand their pharmacological properties.

The human skin covers the entire surface area of the body to protect against pathogens and harmful chemical factors such as solar radiation [50,51]. The organ is composed of several interacting layers: the epidermis, dermis, and subcutaneous tissues. The epidermis covers the outermost side of the skin; 95% of it is composed of keratinocyte, which continuously differentiates to renew and protect injured skin against external pathogens [52]. The epidermis receives nutrients from the dermis [53]. The dermis consists of dermal fibroblasts, which secrete collagen, elastin, and other proteins [54]. Due to the interactions between the functional layers, the skin maintains its strength, flexibility, moisture, and thickness [55]. Over time, different intrinsic and extrinsic factors trigger the skin’s aging process through the degradation of its main components, such as collagen and elastin [56].

Skin aging is a natural process and is mainly categorized as intrinsic or extrinsic aging. Intrinsic aging is stimulated by endogenous factors such as estrogen hormone changes and the mutation of genes [57]. Extrinsic aging is caused by exogenous factors that include smoking, air pollution, and UV exposure [58]. UV exposure mainly causes photoaging, which causes accumulative damage to the functions of skin cells, such as fibroblast ECM synthesis, melanocyte pigment genesis, and Langerhans cell cutaneous immune function [59]. UV radiation provokes excessive pro-inflammatory cytokines, including IFN-γ, ILs, and TNF-α [60]. TNF-α has been studied and shown to bind a specific cell surface receptor, TNFR1. TNF-α causes excessive intracellular ROS generation [61,62]. ROS damage mitochondrial DNA and cause mitochondrial dysfunction in the skin. These lead to the altering of gene expression and accelerate the skin aging process. Additionally, ROS induce various diseases and pathogenic mechanisms such as cardiac disease, cancer, and autophagy [63,64]. Thus, the inhibition of TNF-α-induced ROS generation is a crucial element in preventing skin aging and related diseases. In this study, we investigated the protective effect of LPCE and its chemical constituents on TNF-α-stimulated HDFs. Our results showed that all isolates had an inhibitory effect on ROS generation.

Proinflammatory cytokines trigger the synthesis of matrix metalloproteases (MMPs) such as gelatinase MMP-2, MMP-9, and collagenase MMP-1. Among the MMPs, MMP-1 degrades collagen 1 fibrils, which are the main type of ECM collagen [65]. The LPCE and four flavonol-bis-glucuronides (**1**–**4**) showed a significant inhibitory effect on MMP-1 secretion in TNF-α-induced HDFs. The extract and compounds **1**–**4** significantly decreased MMP-1 secretion. These results suggest that the extract and flavonol-*bis*-glucuronides are potential natural products that protect the skin against various aging factors. The inhibitory effects of the following three flavonol-bis-glucuronides on TNF-α- induced ROS generation and MMP-1 secretion have not yet been reported: isorhamnetin-*bis*-3,7-O-*β*-d-glucuronide (**2**), kaempferol-*bis*-3,7-O-*β*-d-glucuronide (**3**), and quercetin-*bis*-3,7-O-*β*-d-glucuronide (**4**).

Nevertheless, there are possible limitations to this study. For example, TNF-α-induced ROS generation triggers MAPK phosphorylation. This leads to the activation of transcription pathways such as the AP-1 (c-fos and c-Jun) and NF-κB pathways [66]. Through these pathways, an increase occurs in MMP-1 secretion and levels of pro-inflammatory cytokines such as IL-6 and IL-8 [67]. In this study, these pathways have not been presented in detail. To fully understand the protective effects of the *P. chinensis* extract and its isolates, further study is encouraged.

## 5. Conclusions

A new flavonol-*bis*-glucuronide, potentilloside A (**1**)—along with 14 known compounds (**2**–**15**)—were isolated from the leaves of *P. chinensis* in this study. We evaluated the protective effects of the extract and the compounds isolated from *P. chinensis* on TNF-α-stimulated skin aging in HDFs. As a result, the extract and all the compounds tested, including potentilloside A (**1**), inhibited ROS generation in TNF-α-induced HDFs. Furthermore, the extract and flavonol-*bis*-glucuronides (**1**–**4**) diminished MMP-1 secretion in TNF-α-induced HDFs. In particular, potentilloside A (**1**) significantly decreased MMP-1 secretion compared to the TNF-α treatment group. Although further studies should be conducted to understand the mechanisms of anti-skin aging effects in their entirety, these results suggest that extracts from the leaves of *P. chinensis* and potentilloside A (**1**) are candidates to protect skin against photodamage.

## Figures and Tables

**Table 1:** ^1^H and ^13^C NMR spectroscopic data of compounds **1** and **5** (*δ* in ppm, DMSO-*d*_6,_ 500 and 125 MHz).

Position ^a^	1		5 (Quercetin-3-*O*-*β*-d-Glucuronide)	
	*δ*_H_ Multi (*J* in Hz)	*δ*_C_	*δ*_H_ Multi (*J* in Hz)	*δ*_C_
2		155.9		156.3
3		133.3		133.2
4		177.3		177.3
5		161.2		161.3
6	6.23 d (2.0)	98.9	6.21 d (2.0)	98.9
7		164.4		164.3
8	6.48 d (2.0)	94.0	6.41 d (2.0)	93.7
9		156.4		156.4
10		104.0		104.0
1′		121.0		120.9
2′	7.75 d (2.5)	116.8	7.55 d (2.5)	116.2
3′		144.6		145.0
4′		150.2		148.7
5′	6.92 d (8.5)	115.9	6.84 d (8.5)	115.3
6′	7.89 dd (8.5, 2.5)	125.5	7.60 dd (8.5, 2.5)	121.8
Glu-1″	5.46 d (7.5)	101.2	5.49 d (7.5)	101.2
Glu-2″	3.368 *^b^*	73.1	3.24–3.40 m ^b^	73.9
Glu-3″	3.244 *^b^*	75.8	3.24–3.40 m ^b^	76.0
Glu-4″	3.370 *^b^*	71.4	3.24–3.40 m ^b^	71.5
Glu-5″	3.55 d (10.0)	75.9	3.57 d (10.0)	76.0
Glu-6″		170.3		170.0
Glu-1‴	5.08 d (7.5)	101.5		
Glu-2‴	3.240 *^b^*	73.8		
Glu-3‴	3.365 *^b^*	75.3		
Glu-4‴	3.43 t (9.5)	71.4		
Glu-5‴	3.94 d (9.5)	75.4		
Glu-6‴		170.1		
OH-5	12.52 s		12.54 s	

^a^ Determined by analysis of ^1^H-^1^H COSY, ^1^H-^13^C HSQC, and HMBC spectrum, ^b^ Overlapped.

## Data Availability

Not applicable.

## Supplementary Materials

The following supporting information can be downloaded at: https://www.mdpi.com/article/10.3390/plants11233318/s1, Figure S1: HR-ESI-Orbitrap-MS spectrum of compound **1**; Figure S2: The IR spectrum of compound **1**; Figure S3: The ^1^H-NMR spectrum of compound **1** (500 MHz, DMSO-*d*_6_); Figure S4: The ^13^C-NMR spectrum of compound **1** (125 MHz, DMSO-*d*_6_); Figure S5: The HSQC spectrum of compound **1**; Figure S6: The COSY spectrum of compound **1**; Figure S7: The HMBC spectrum of compound **1**; Figure S8: The ROESY spectrum of compound **1**.
