# Supplementary material for: Potentilloside A, a New Flavonol-bis-Glucuronide from the Leaves of Potentilla chinensis, Inhibits TNF-α-Induced ROS Generation and MMP-1 Secretion
Source: Plants (Basel). 2022 Dec 1;11(23):3318. doi: 10.3390/plants11233318 (PMC9738723; doi:10.3390/plants11233318)

Supporting information

Potentilloside A, a New Flavonol-*bis*-glucuronide from the Leaves of *Potentilla chinensis*, Inhibits TNF-α-Induced ROS Generation and MMP-1 Secretion

Contents of supporting information

Figure S1. HR-ESI-Orbitrap-MS spectrum of compound **1**.

Figure S2. The IR spectrum of compound **1**.

Figure S3. The ^{1}H-NMR spectrum of compound **1** (500 MHz, DMSO-d_6).

Figure S4. The ^{13}C-NMR spectrum of compound **1** (125 MHz, DMSO-d_6).

Figure S5. The HSQC spectrum of compound **1**.

Figure S6. The COSY spectrum of compound **1**.

Figure S7. The HMBC spectrum of compound **1**.

Figure S8. The ROESY spectrum of compound **1**.

Figure S1. HR-ESI-Orbitrap-MS spectrum of compound **1**.

Elemental Composition Results - Spectrum 1

Peak Mass	Display Formula	S Fit	RDB	Delta [ppm]	Theo. mass	Rank	Combined Score	# Matched Iso.	# Missed Iso.	MS Cov. [%]	Pattern Cov. [%]
655.1142	$C_{27}H_{27}O_{19}$	54.52	14.50	0.14	655.11410	1	97.49	5	0	99.88	99.82

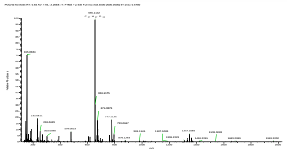

Figure S2. The IR spectrum of compound **1**.

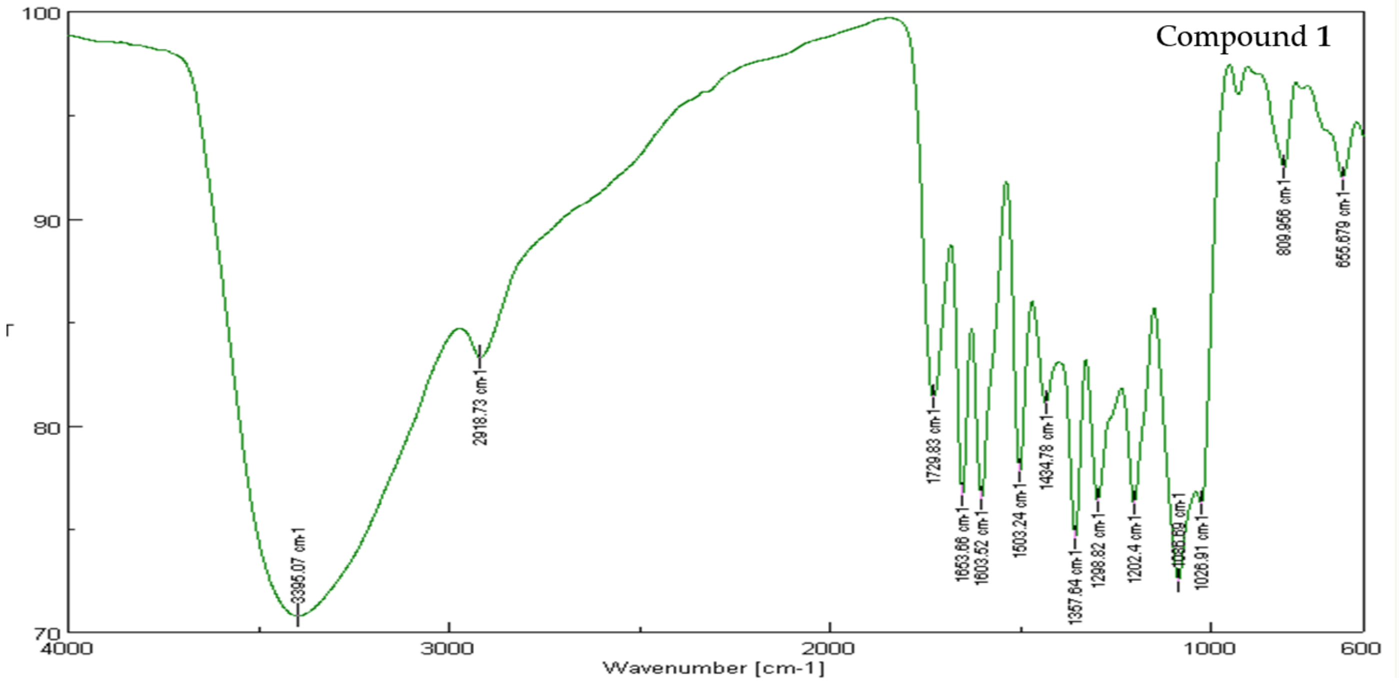

Figure S3. The ^{1}H-NMR spectrum of compound **1** (500 MHz, DMSO-d_6).

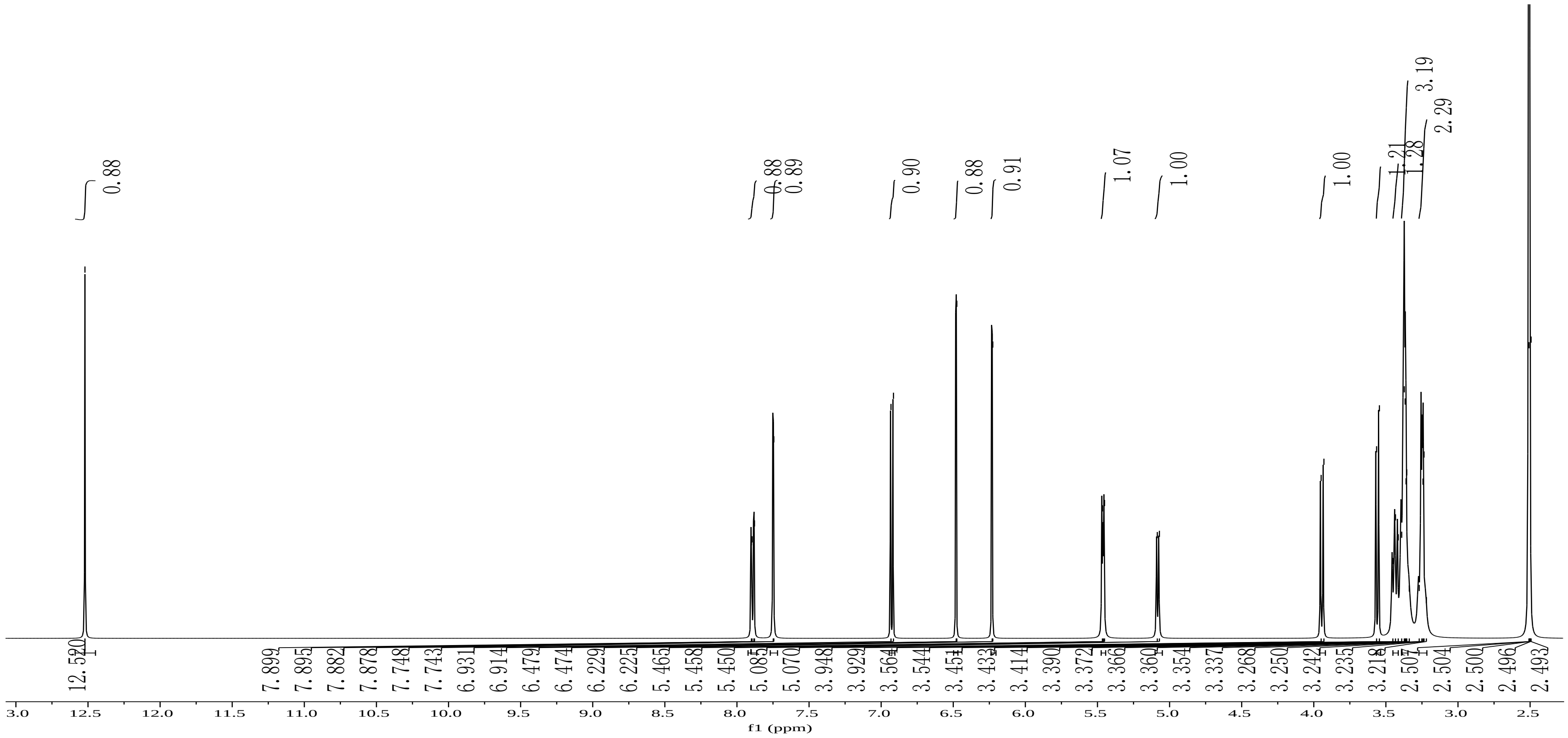

Figure S4. The ^{13}C-NMR spectrum of compound **1** (125 MHz, DMSO-d_6).

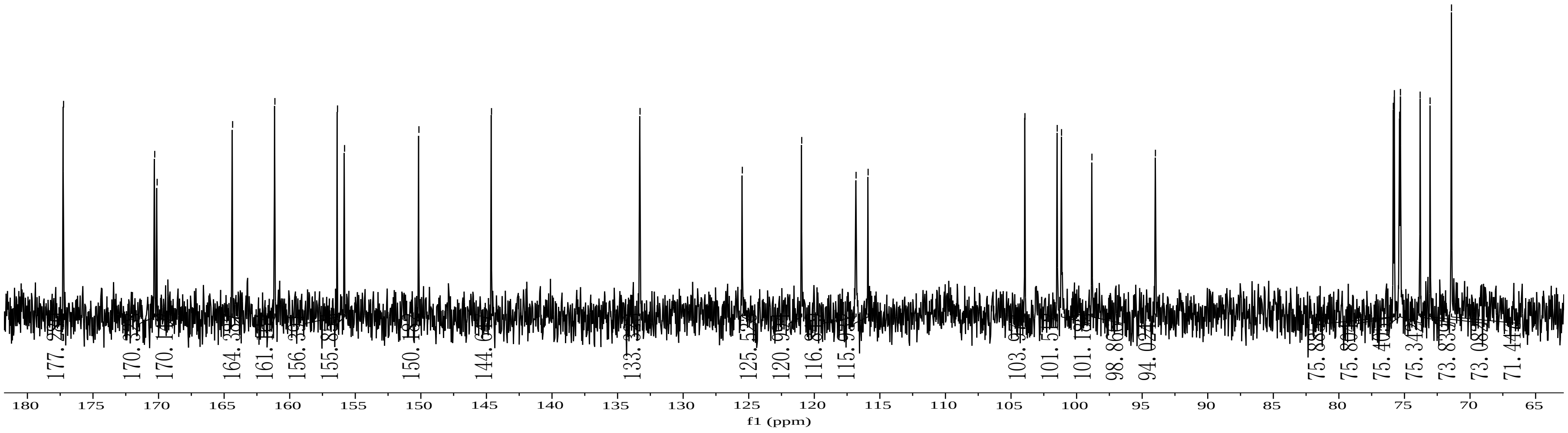

Figure S5. The HSQC spectrum of compound **1**.

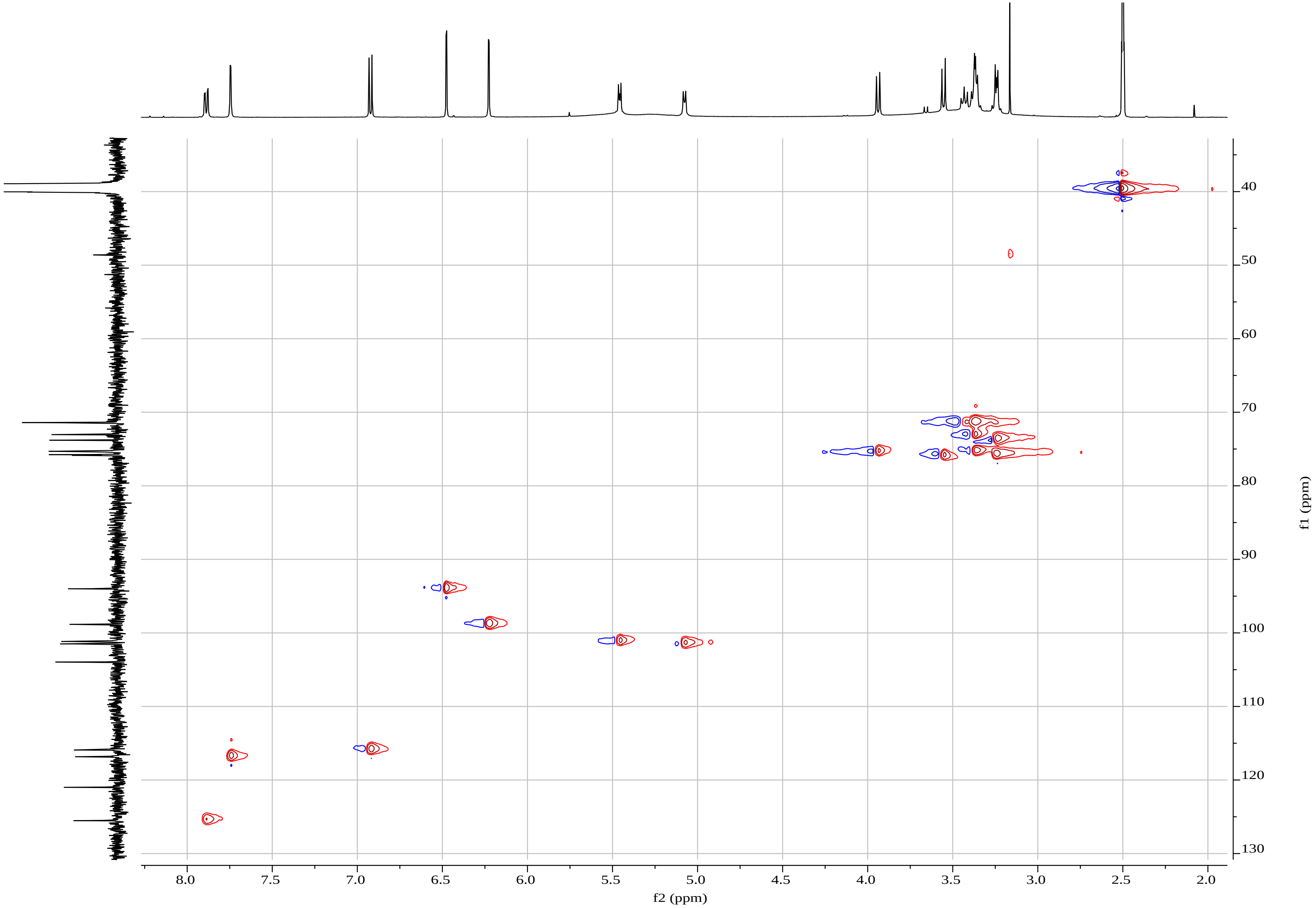

Figure S6. The COSY spectrum of compound **1**.

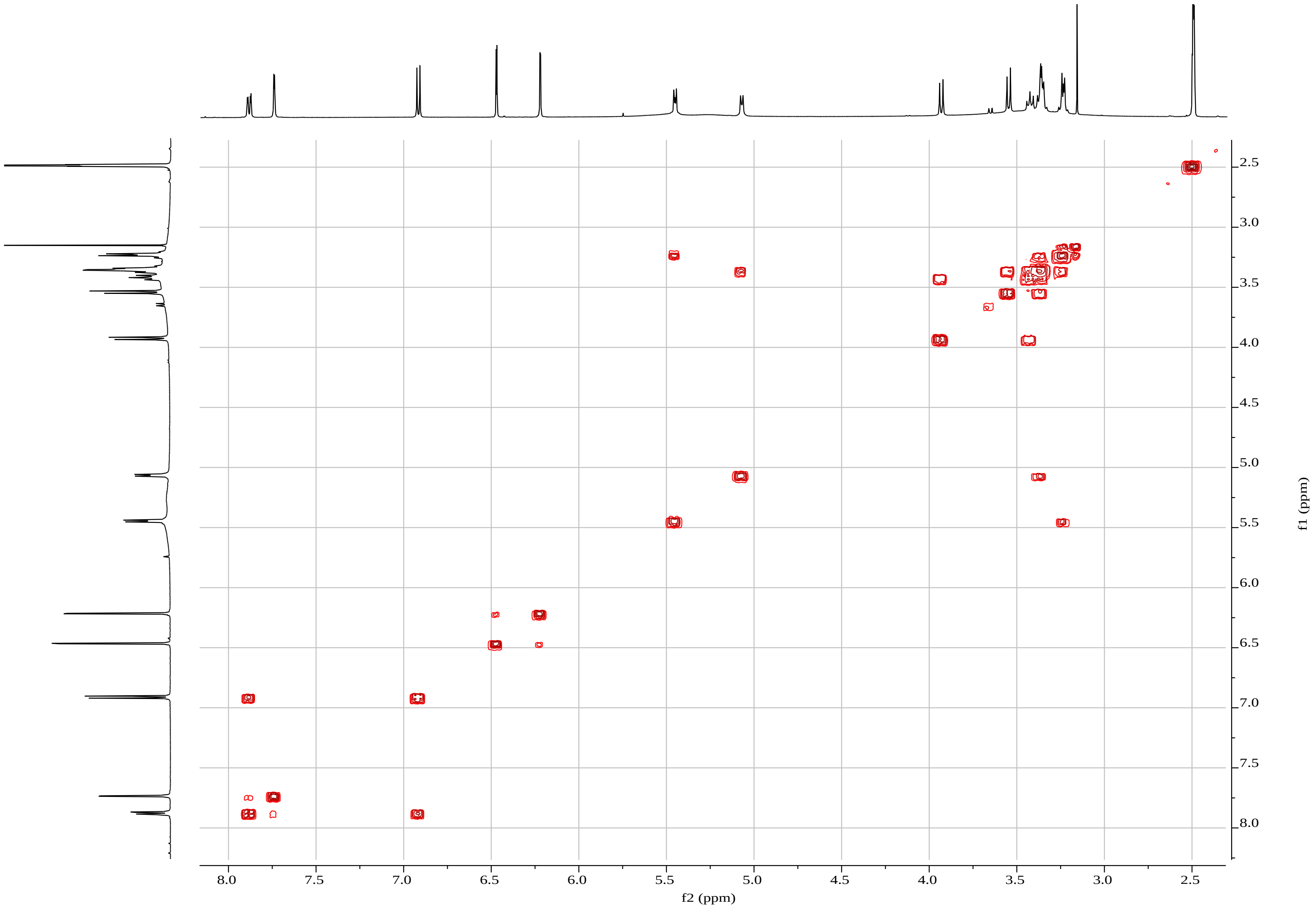

Figure S7. The HMBC spectrum of compound **1**.

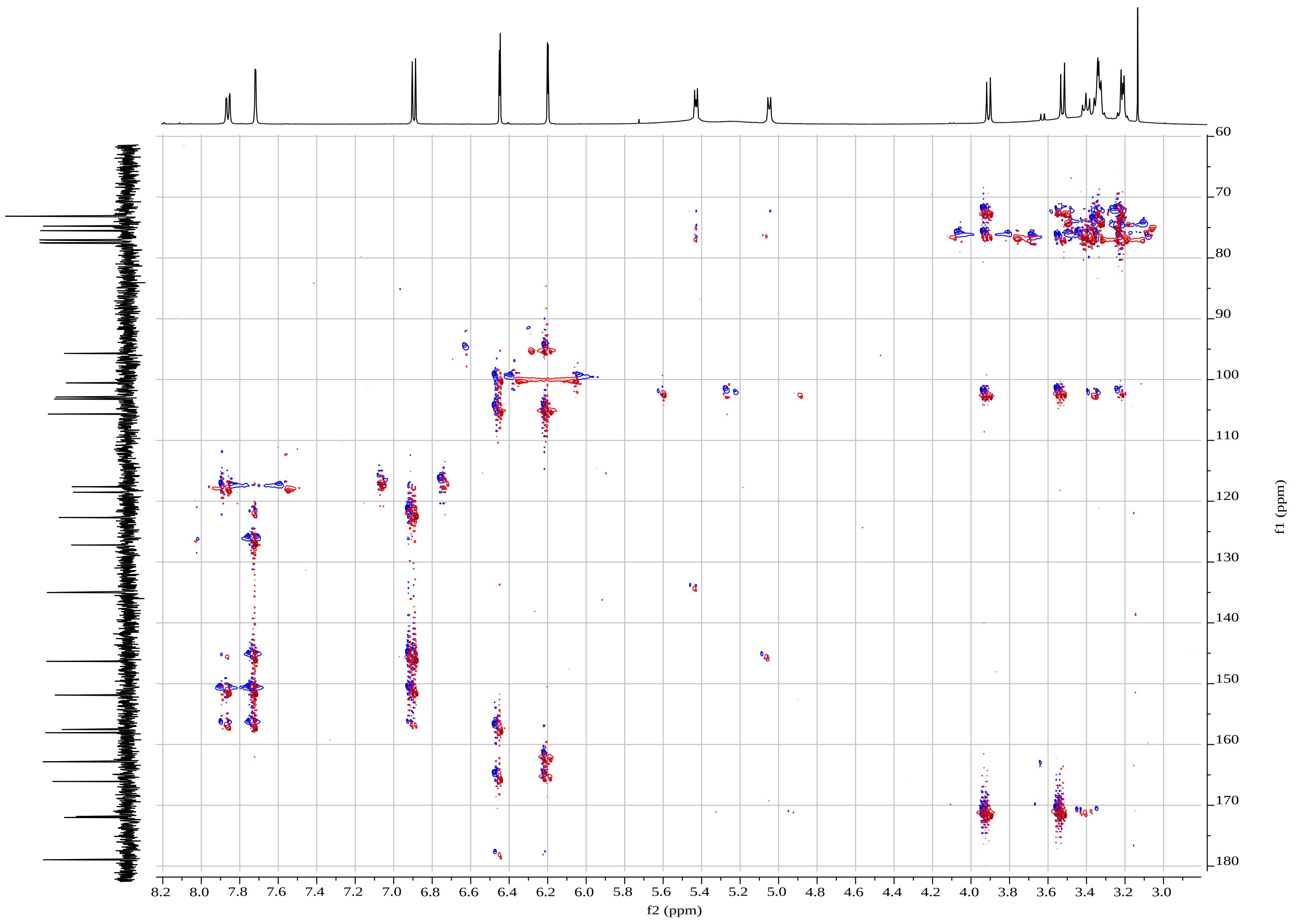

Figure S8. The ROESY spectrum of compound **1**.

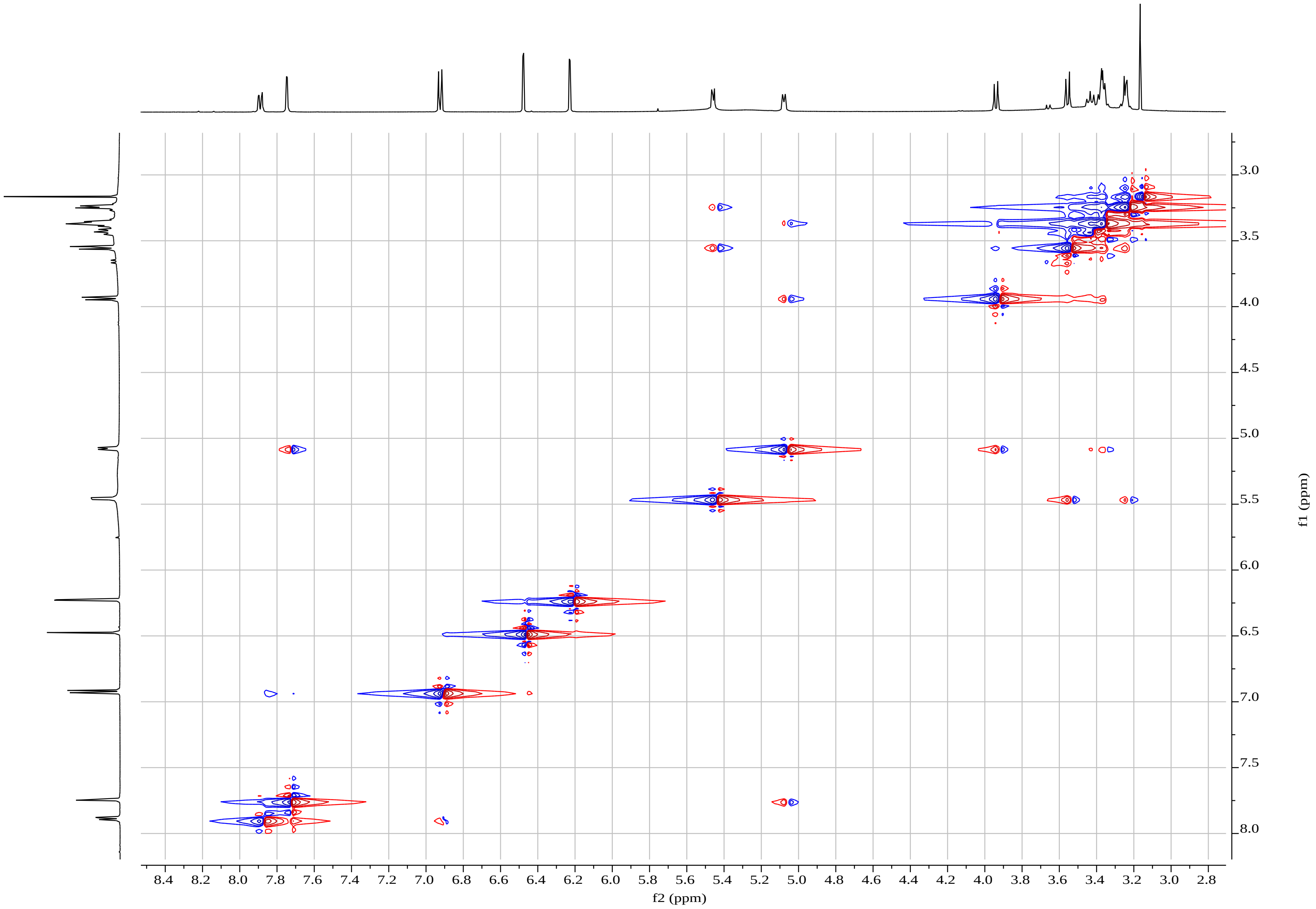

Supplement: Supplementary file 1 [file plants-11-03318-s001.zip › plants-2034460-supplementary.pdf]
